# Measurement of availability and accessibility of food among youth: a systematic review of methodological studies

**DOI:** 10.1186/s12966-017-0477-z

**Published:** 2017-02-14

**Authors:** Mekdes K. Gebremariam, Cristina Vaqué-Crusellas, Lene F. Andersen, F. Marijn Stok, Marta Stelmach-Mardas, Johannes Brug, Nanna Lien

**Affiliations:** 1 Department of Nutrition, Faculty of Medicine, University of Oslo, PO Box 1046, Blindern, N-0316 Oslo, Norway; 2 Faculty of Health Science and Wellbeing, University of Vic-University Central of Catalonia, Barcelona, Spain; 3 Department of Psychological Assessment and Health Psychology, University of Konstanz, Konstanz, Germany; 4 Department of Epidemiology, German Institute of Human Nutrition Potsdam-Rehbruecke, Nuthetal, Germany; 5 Department of Pediatric Gastroenterology and Metabolic Diseases, Poznan University of Medical Sciences, Poznan, Poland; 6 Amsterdam School of Communication Research, University of Amsterdam, Amsterdam, The Netherlands

**Keywords:** Availability, Accessibility, Measurement, Reliability, Validity, Conceptualization, Youth

## Abstract

**Background:**

Comprehensive and psychometrically tested measures of availability and accessibility of food are needed in order to explore availability and accessibility as determinants and predictors of dietary behaviors. The main aim of this systematic review was to update the evidence regarding the psychometric properties of measures of food availability and accessibility among youth. A secondary objective was to assess how availability and accessibility were conceptualized in the included studies.

**Methods:**

A systematic literature search was conducted using Medline, Embase, PsycINFO and Web of Science. Methodological studies published between January 2010 and March 2016 and reporting on at least one psychometric property of a measure of availability and/or accessibility of food among youth were included. Two reviewers independently extracted data and assessed study quality. Existing criteria were used to interpret reliability and validity parameters.

**Results:**

A total of 20 studies were included. While 16 studies included measures of food availability, three included measures of both availability and accessibility; one study included a measure of accessibility only. Different conceptualizations of availability and accessibility were used across the studies. The measures aimed at assessing availability and/or accessibility in the home environment (*n* = 11), the school (*n* = 4), stores (*n* = 3), childcare/early care and education services (*n* = 2) and restaurants (*n* = 1). Most studies followed systematic steps in the development of the measures. The most common psychometrics tested for these measures were test-retest reliability and criterion validity. The majority of the measures had satisfactory evidence of reliability and/or validity. None of the included studies assessed the responsiveness of the measures.

**Conclusions:**

The review identified several measures of food availability or accessibility among youth with satisfactory evidence of reliability and/or validity. Findings indicate a need for more studies including measures of accessibility and addressing its conceptualization. More testing of some of the identified measures in different population groups is also warranted, as is the development of more measures of food availability and accessibility in the broader environment such as the neighborhood food environment.

## Background

The promotion of healthy dietary behaviors among youth is pivotal for the prevention of overweight/obesity as well as non-communicable diseases [1, 2]; dietary behaviors learned in childhood are also found to track into adulthood [3]. To develop effective interventions targeting different dietary behaviors, it is imperative to understand the important correlates of these behaviors. Examples of such correlates include the availability and accessibility of food, as well as other factors such as self-efficacy, food preferences, parental modeling and parental rules. Availability and accessibility of foods as potential determinants of food choice and dietary intake are recognized in most up-to-date theories aiming to predict and/or explain health behaviors including dietary behaviors. For example, social–ecological theories of health behavior [4] posit that the physical and social environment we live in importantly influences our health behaviors. The food environment children and adolescents live in –especially the home and school environments- define what foods are available and accessible to them. A further detailing and specification of social cognitive theory for energy balance-related behaviors including dietary behavior, was proposed by Kremers et al. [5]. They argue that the influence of such environmental factors –including availability and accessibility- may be mediated and moderated by individual level, social and demographic determinants such as intentions, preferences, self-efficacy, and social environmental factors such as socioeconomic position, parenting, and modeling. Most major theoretical models aiming to explain food choice and dietary behaviors nowadays directly or indirectly recognize the importance of and interplay between physical environmental factors -such as availability and accessibility of foods-, social environmental factors and personal factors as important drivers of food choice and dietary behavior, which is why further insight in and overview of the measurement qualities of measures to assess these issues, is of importance. However, existing evidence suggests that there is a high variation in the conceptualization of correlates and determinants of dietary behaviors, as well as a common use of measurement instruments whose psychometric properties are not tested [6, 7]. These issues are problematic for several reasons. The first is the difficulty to identify important determinants of dietary behaviors due to the presence of significant measurement errors. These errors might be particularly pronounced in studies involving children due to varying cognitive development that might affect comprehension and recall of the construct in question. The second is the inability to compare findings across different populations and settings when different measures are used to explore the same correlate.

Availability and accessibility of foods are among the correlates most consistently associated with dietary behaviors among youth [8–11]. In addition, their importance in explaining socioeconomic differences in dietary behaviors has been evidenced by several studies using formal tests of mediation [12–14]. However, the conceptualization of these constructs has not always been uniform, in particular in relation to the concept of accessibility of foods. Availability is related to the physical presence of food; this can include foods offered/served in different settings. In a recent Delphi study aimed at clarifying food parenting practices related to snacking, different descriptions were given by experts in relation to availability including having food at home, offering food, serving food and making sure foods are prepared [15]. Accessibility on the other hand has been defined as reflecting whether foods are available in a form and location that facilitate their consumption [16]. The need for the food to be retrievable and ready to eat has also been highlighted [17]. In the aforementioned Delphi study, the following descriptions were put forward by experts as being related to the accessibility of snacks: “storing snacks in a location the child cannot access on his or her own”, “not giving the child money for snacks at school”, “avoiding going to shops where snacks are available”, “putting snacks on the table all day” etc. [15]. These conceptualizations show the different dimensions of these constructs and the need to consider these while looking at instruments measuring these concepts and while summarizing evidence related to the role of these correlates in influencing dietary behaviors.

Previous reviews have looked at measurement properties of correlates of dietary behaviors among youth including availability and/or accessibility of food [18–20]. Findings indicate that several measures of availability and accessibility of food, in particular related to the home environment, do exist. While evidence of reliability exists for several of these instruments, a lack of validity assessment was documented across reviews. The present review includes studies published from 2010 onwards not included in previous reviews of studies exploring the psychometric properties of measures of the availability and/or accessibility of food among youth. Unlike the previous reviews, the focus is only on methodological studies, and, in addition to summarizing the psychometric properties of the instruments, the review will also describe differences in the conceptualization of these correlates across studies. Providing such an overview of existing measures and their psychometric properties will help to avoid unnecessary replication of existing measures and help identify gaps in the measurement of the constructs of interest.

## Methods

### Search strategy

The systematic steps outlined in the PRISMA guidelines were used in this review [21]. The studies of interest were those reporting on the psychometric properties of measures assessing the availability and/or accessibility of foods/drinks among youth. The location could be at home, at school and in the neighborhood (e.g. stores). Therefore, the search was conducted by combining, using the “AND” Boolean operator, five main groups of keywords: keywords for dietary behaviors (e.g. food habits, dietary habits, dietary behavior), keywords for the correlates (e.g. availability, accessibility), keywords for psychometric properties (e.g. validity, reliability), keywords for methods used (e.g. survey, questionnaire) and keywords for the population of interest (e.g. children, adolescents, youth). Within each of these categories, keywords were combined using the “OR” Boolean operator. The search strategy is available from the corresponding author upon request. The following databases were searched for relevant articles using keywords and Medical Subject Headings: Medline, Embase, PsycINFO and Web of Science. In addition, reference lists of relevant publications were manually searched.

### Inclusion criteria

The following inclusion criteria were used in this review: i) methodological studies where the aim/one of the aims of the study is to evaluate at least one measurement property of an instrument measuring accessibility and/or availability of food, ii) the measurement instrument relates to the availability and accessibility of food in one or more of different food environments of youth (0–18 years), iii) studies published in English in peer-reviewed journals, iv) studies published between January 2010 and March 2016.

### Identification of relevant studies and data extraction

Titles and abstracts of retrieved studies were screened to assess whether inclusion criteria were met. When the abstract was considered insufficient to make conclusions about inclusion, the full text was screened. A standardized form for extraction of detailed data from each included study was developed. The data extracted included study and sample description (including year of study publication, country, age of participants, gender composition, sample size, socioeconomic background (when available)). Information about the measures used was also extracted and included the type of construct assessed (availability and/or accessibility), the name (if any) of instrument, the type of instrument (e.g. self-report questionnaire, inventory, observation form), the number of items included and the methods for item development.

Information on the conceptualization of availability and accessibility was extracted, when explicitly presented in the studies. Information regarding internal consistency, test-retest and inter-rater reliability, as well as content (including face), construct and criterion validity was also extracted when and where available. Responsiveness, which refers to the sensitivity of the measure for the assessment of change [22], was also of interest. Results of test-retest and inter-rater reliability, often expressed as correlation coefficients, were interpreted using Landis & Koch’s criteria: slight (*r* = 0.00–0.19); fair (*r* = 0.20–0.39); moderate (*r* = 0.40–0.59); substantial (*r* = 0.60–0.79); and almost perfect (*r* = 0.80–1.0) reliability. Kappa values were similarly interpreted [23].

Internal consistency reliability was defined as adequate when Cronbach’s alpha coefficient above 0.6 was reported [24]. It was also considered adequate if exploratory factor analysis was conducted [25].

Face and content validity: face validity is an aspect of content validity and involves a subjective assessment with no specific standards as to how it should be assessed and cannot be quantified [22]. When the study sample was clearly described, and a clear conceptualization of the measure was provided or a previously validated instrument was used or the item development and refinement is clearly presented, the instrument was described as having face validity. Use of independent experts is an aspect of the assessment of content validity [22]; instruments in studies where experts were used in the development or refinement phase of development were therefore considered to have content validity.

Criterion validity and construct validity can be quantified using different parameters such as correlation coefficients or percentage of agreement values. For these constructs, a correlation of 0.3 or above was considered acceptable [26, 27]; a correlation of above ≥ 0.7 was considered very good [27]. When percentage agreement values were used, agreement levels were categorized as follows: “good to excellent” (>75%), “moderate” (60–74%), or “poor” (< 60%) [28].

### Assessment of study quality

In addition to the quality criteria for the assessment of reliability and validity described above, assessment of the quality of the methods of item development and refinement was done.

The assessment was based on how systematic the process of item development was, including the methods that were used in the process of item development (e.g. use of items from existing instruments, use of expert opinion, use of theory, use of existing literature, use of qualitative methods etc.). It also included an assessment of whether any method was used for item refinement (e.g. pilot testing, cognitive interviews or use of experts). The following scores were given: 4 = fully systematic process of item development and use of at least one method of item refinement; 3 = fully systematic process used for item development but no method reported for item refinement OR process not fully systematic but item refinement was done; 2 = process of item development was not fully systematic and no method reported for item refinement; 1 = no systematic process was reported for the development or refinement of items. This grading was modified (to fit the type of studies included in the present review (i.e. methodological studies only) and the constructs assessed) from the grading developed by Vaughn et al. [29].

Two researchers (MKG, CV) independently extracted data and assessed study quality; discrepancies were resolved through discussion.

## Results

**Fig. 1:**
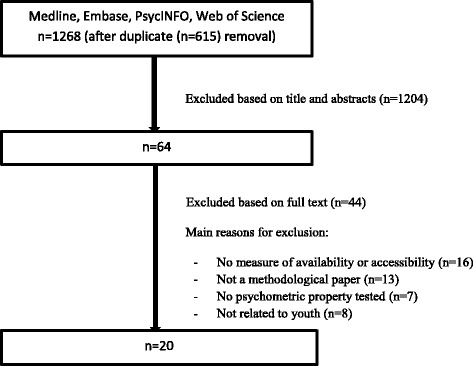
Flowchart indicating the steps followed in the literature search

The literature search yielded a total of 1268 potentially relevant papers after removal of duplicates. After a review of titles, abstracts and full-texts, 20 studies that met the inclusion criteria were found [30–49]. No additional studies were found through manual reference searching (Fig. 1).

### Characteristics of included studies

Table 1 (columns 1–3) describes the characteristics of the included studies. Most of the studies were conducted in North America (*n* = 13); the rest were conducted in Australia (*n* = 3), Europe (*n* = 2), China (*n* = 1) and Brazil (*n* = 1). The target population ranged from pre-school children to adolescents. The sample size varied between 13 [37] and 730 [45]. Participants and settings from different socioeconomic backgrounds were included in most of the included studies. In four of the studies where information was provided by parents, the sample was predominantly made up of mothers [33, 34, 37, 48]. In the studies where children or adolescents provided information, both male and female participants were included. In the studies where ethnic composition was reported [30, 31, 33, 35, 37, 43, 44, 46–48], participants with different ethnic backgrounds were included although there was a variation in the proportion of participants from different ethnic groups; one study included ethnic minorities only [35]. The food environment in which these instruments aimed to assess availability or accessibility included the home environment (11 studies) [30–35, 37, 38, 44, 45, 48], the school (4 studies) [39, 40, 43, 47], stores (3 studies) [36, 42, 49], childcare/early care and education services (2 studies) [41, 46] and restaurants (1 study) [49]. While 15 [30, 31, 34, 36, 39–49] studies included measures of food availability only, three included measures of both availability and accessibility [35, 37, 38] and one included a measure of accessibility only [32]. Two studies included two different measurement instruments [37, 43]. One of the instruments used by Nepper et al. [37] was adapted from the instrument developed by Boles et al. [38]. Different types of foods and beverages were included in the studies. However fruits and vegetables were the most commonly included foods.

**Table 1:** Description of studies and instruments included in the review

Author, year, country	Sample description (sample size, age, gender distribution)	Construct assessed	Name of instrument	Instrument type (self-report, interview etc)	Number of items	Methods for item development (grading 1–4)
Boles et al., 2014, US [30]	83 caregivers of preschool children ((51% female), 48.1 (19.2) months), 57% aged 30–49, 89% high school diploma or less, 22% hispanic, 47% with incomes < $27,000. Trained researchers conducted observations on 25 randomly selected homes (rater-parent inter-rater reliability study)	Availability of different food items categorized as snacks, cereals, drinks, fruits and vegetables, meats, dairy, breads, ready to eat meals and others in the home	Home-IDEA (Home Inventory Describing Eating and Activity Development)	Home inventory	131 initially, 62 found to be unacceptable based on κ values (comparing observer-parent reports) and removed (categorized into 9 food categories)	Based on a previously validated instrument, new items added (to capture foods potentially consumed by families with geographical and SES diversity) based on existing food frequency questionnaires and an exisiting allowable foods list, further items added based on expert opinion. Items removed after assessment of criterion validity- score = 4
Dewar et al., 2012, Australia [31]	173 secondary school students, age: 13.7 (1.2), 62% female, 80% Australian	Availability of healthy snacks, healthy drinks, fruit and vegetables in the home and in general		Self-report questionnaire	6 initially reduced to 4	Qualitative methods used to develop and refine the scale; literature review was also used; experts were consulted to among other things assess content validity; focus groups were then conducted to further review and refine scales and after changes were made, expert panel was again asked to review the scales - score = 4
Bennaroch et al., 2011, Spain [32]	591 students aged 15–16 years, 50% girls, 61% from public schools, 37% had only the male parent working	Accessibility of fish, fruit and vegetables at home and ability to eat everything and in reasonable amount	Food consumption, intentions and preferences assessment test- FCIPAT questionnaire	Self-report questionnaire	3 items	Reviews of previous surveys on adolescent dietary habits and their correlates led to the first version of the questionnaire; the items were assessed by experts and changes made. Finally, a pilot study was conducted among students of same age and items were further refined - score = 4
Vyduna et al., 2016, US [33]	Parents of young adolescents, *n* = 166, 87% female, 88% between 31 and 50 years, 61% Hispanic, 54% with college degree; 71 parents included in test-retest study	Availability of calcium-rich foods in the home		Self-report questionnaire	10 items	Open-ended person interviews were conducted with parents of young adolescents to identify factors to be included; the social cognitive theory was used to define the constructs and constructs’ subscales in the overall questionnaire; cognitive interviewing was then used to evaluate the first drafts of the questionnaire; finally, field testing was conduted, content validity was assessed by a group of experts, score = 4
Petty et al., 2013, Brazil [34]	Parents of young children (mean age 8.3 (1.2), 52% girls), 582 parents, mostly mothers (86%), 71% of mothers and 60% of fathers had finished college; 55 participated in test-retest and 58 in convergent validity test	Availability of fruits and vegetables at home	Parent Mealtime Action Scale (PMAS), Portuguese version	Self-report questionnaire	3 items	The authors aimed to validate the PMAS which was previously validated using American parents, score = 2
Hearst et al., 2012, US [35]	30 low income, non-English speaking families (Somali and Hispanic) with children of pre-school age; only 3 had some college or graduate degree and only 4 had an annual household income of greater than 30,000 USD per year	Availability of dairy, vegetables and fruits, meats and other non-diary protein, added fat, frozed desserts, prepared desserts, savoury snacks, micorwavable/quick-cook foods, bread, candy, cereals and beverages at home. Accessibility of selected foods at home	Home Food Inventory (HFI) translated into Somali and Spanish	Checklist	12 food categories with subcategories for availability, 1 item on obesogenic food environment, 4 items for accessibility	Existing HFI was used and focus groups were used to modify the form as it relates to potential cultural food preferences; modifiations were then made - score = 3
Rimkus et al, 2013, US [36]	120 stores located in low, middle and high income tracts, and within and outside city limits were surveyed by 6 trained observers	Availability of healthy and unhealthy food items and beverages in stores, number of fruit and vegetable options available	Bridging the gap - Food store observation Form (BTG-FSOG	Observation form	56 items	Initially developed building on existing instruments and inputs from researchers, practitioners and advodates with expertise in nutrition, measurement of the food environment and food policy, then pre-tested and modified - score = 4
Nepper et al, 2014, US [37]	13 pairs of parents and their children aged 9-12 years (instruments filled in by parents); mean age of parents was 40.2 (4.9), the majority were mothers, 9 parents were college graduates and 9 were non-hispanic white, 11 had an income of 50,000 dollars or more	Availability and accessibility of healthy and unhealthy food items, and of fruits and vegetables separately at home, HFA instrument adapted from Boles et al., 2013	Home Food Assessment Tool (HFA) and 30-day Home Food Environment Survey (HFES)	Self-report inventory and survey instrument	HFA: 23 items (healthy foods and beverages, unhealthy foods and beverages); 18 items (fresh fruits); 14 items (fresh vegetables); HFES: 18 items	Both instruments were modified from previously validated instruments - score = 2
Boles et al., 2013, US [38]	Trained research assistants conducted the survey in homes, 82 families of preschool children (mean age of 50.9 months); 35 obese and 47 healthy weight children, majority had income between 50,000–124,000 USD; for the inter-rater reliability, 18 observers included	Availability and accessibility of 23 healthy and unhealthy food items and of fruits and vegetables at home	Home Health Environment (HHE) instrument	Observation tool	23 food items (healthy foods and beverages, unhealthy foods and beverages); 18 items (fresh fruits), 14 items (fresh vegetables)	Previously validated self-report instruments (with good construct and test-retest reliability) provided the preliminary item pool, experts were consulted to further refine items and operationalize definitions - score = 4
Nathan et al., 2013, Australia [39]	42 primary school principals; 57% of schools were medium size, 67% were government schools, 98% were urban and 67% were in higher socioeconomic areas	Availability of healthy and unhealthy food items at school via a) canteen, b) vending machines, c) via fundraisers	School Environment Assessment Tool (SEAT)	Computer assisted telephone survey	26 items (vending machine), 13 items (canteen), 13 items (fund raising)	The instrument was developed by conducting a systematic review of the literature, followed by a draft survey which was assessed by experts, further refined and pilot tested among primary school principals to check for acceptability and comprehension - score = 4
Lee et al., 2014, US [40]	Directors from 35 afterschool programs in 3 cities (step 1 conducted in 20 programs, and after revisions step 2 was conducted in 15 programs), low income, racially diverse settings	Availability of fruit, vegetable, grains, whole grains, water, 100% juice in after-school settings	Observations of physical activity participation and snack consumption (OSNAP-OPAT)	Observation tool	5 items (fruit or vegetable, grains, whole grains, water, 100% juice)	Items were focused on measuring specific intervention goals; the tool was piloted during after-school program time, reviewing the usefulness of instructions and clarity and feasiblity - score = 3
Dodds et al., 2014, Australia [41]	Nominated supervisors and room leaders of 42 childcare services (preschools and long day care services), medium SEIFA for 69%, majority of services were long day care services (62%), 88% open 5 days	Availability of cordial, flavoured milk, fruit juice, water, plain milk, soft drink, healthy foods, confectionary, chocolate, ice-cream, fruit or vegetable pieces, salads or platters, pretzels, plain popcorn or oven-baked chips, sweet biscuits with chocolate or cream filling in childcare services		Self-report survey instruments	14 items (8 beverages and 6 foods)	Survey items were based on literature review and on existing tools, together with surveys previously developed and implemented by the research group, as well as regulations about service policies and practices related to healthy eating, obesity etc., surveys were piloted to assess comprehension and understanding, and items were then amended score = 4
Izumi et al., 2014, US [42]	Trained observers conducted the study in 50 food stores located near elementary and middle schools	Availability of healthier alternatives to energy-dense snacks and beverages consumed by children in food stores	SNACZ food store checklist	Checklist	48 snacks and beverages (6 beverages, 18 snacks, 24 fresh and frozen vegetables)	The checklist was developed using a multi-step process, with snack and beverage items consumed by children identified by reviewing relevant literature and tools and surveying more than 750 children. The identified items were pretested in 10 food stores, score = 4
Krukowski et al., 2011, US [43]	Trained raters completed the instrument at 81 schools, 58% elementary, 61% children eligible for free/reduced lunch, 73% white children, 42% medium sized	Availability of fruits, vegetables, grains, side dishes, entrees, chips, desserts, a la carte, beverages in public school cafeterias	SCNA-O (to observe foods offered in school cafeterias) and School Cafeteria Nutrition Assessment (SCNA)-M (to evaluate monthly school lunch menus)	Observation tool and menu assessment form	9 broad categories of foods/beverages	School menus from across the US were reviewed, school lunches were observed, and related food availability measures were evaluated; the items were then pre-tested and revisions were made; a pilot study was also conducted to assess the feasibility and reliability of the SCNA and modifications to the rater instruction manual were made, score = 4
Ding Ding et al., 2012, US [44]	Adolescents (age: 14.6 (1.7), 51% females, 53% non-hispanic white), children (age: 8.3 (1.9), 52% females, 78% non-hispanic white), parents (age: 39.6 (7.7), 85% females) (*n* = 458), median household income $ 60,000–69,999	Availability of fruits and vegetables, more-healthful food, less-healthful food at home		Self-report questionnaire	19 items (3 for availability of fruits and vegetables, 7 for availability of more-healthful foods, 9 for availability of less-healthful foods)	Instrument developed based on ecological model and on a list of food items recommended by other authors - score = 3
Singh et al., 2011, six European countries (Belgium, Greece, Hungary, the Netherlands, Norway, Spain) [45]	10-12 year old children (*n* = 730 for test-retest reliability and *n* = 96 for construct validity). For test-retest study, number ranged from 86 (Spain) to 155 (Greece), number ranged between 15 and 20 per country for construct validity study	Availability of fizzy drinks or fruit squash, fruit juice and breakfast products at home	ENERGY-child questionnaire	Self-report questionnaire	3 items	The questionnaire was developed based on existing validated questionnaire used in different European settings, the availability items were taken from the pro-children study; pre-tested among small samples in all participating countries to examine comprehensibility and duration of completion, score = 3
Ward et al., 2015, US [46]	Teachers (average age was 37 years, 28% had bachelor’s degree or higher) in 50 early care and education centers provided information over 4 days. The centers had 52% of enrolled children who received subsidies towards their enrollment fees, and 57% were hispanic	Availability of foods and beverages (total grains, meat or alternative, fruit, vegetables, milk, 100% fruit juice, water) in early care and education centers - called serving in the paper	Staff Daily Questionnaire, which is one of the surveys of the Environment and Policy Assessment and Observation- Self report (EPAO-SR) instrument	Self-report questionnaire	7 categories of food items	Instrument developed in different phases: modification of items from existing observation-based instrument; review by community advisory group and experts; cognitive interviews with center directors and classroom teachers. Revisions were subsequently made, score = 4
Anzman-Frasca et al., 2015, US [47]	Program leaders from 65 OST (Out-of -School-Time) programs; 1st sample (*n* = 31 programs), mean number of children = 29, <5% were < 5 years and 50% were 8–12 years, 45% girls, 50% white, 48% traditional afterschools; 2nd sample (*n* = 34 programs), mean number of children = 11, <1% were < 5 years and 77% were 8–12 years, 32% girls, 95% white, 91% enrichment programs	Availability of foods and beverages (fresh FV, processed FV, salty snacks, sweet snacks, protein snacks, water, milk, juice, sweetened beverages) in outof-school-time programs	Out-of -School-Time Snacks, Beverages and Physical Activity Questionnaire (OST-SBPA)	Self-report questionnaire	9 categories of foods and beverages	A team of nutrition researchers developed questionnaire; grouping made based on categories created by reviewing categories used in national surveillance datasets, snacks observed in field studies of OST and childcare programs, and evidence linking snacks and beverages with energy intake and weight status. Pilot testing was then done in a separate group of program leaders, score = 3
Fulkerson et al., 2012, US [48]	51 adults, mean age 39.4 (7.0), 94% female, 68% white, 62% had a college degree	Availability of foods at home meals (served at meals): meat or other protein, beverages, vegetables, other starch, dessert, bread, salad, fruits		Self-administered screening instrument	8 categories of foods assessed (with sub-categories)	Initial list of items drafted by investigators, opinions from experts requested for further instrument development and assessment of face validity. The instrument was then revised. Field testing was conducted to assess ease of completion and identify foods difficult to include on the form, score = 4
Hua et al., 2014, China [49]	Two pairs of trained researchers assessed 141 restaurants and 84 retail food stores in three neighborhoods that vary in real estate prices, development histories and land use characteristics	Availability of basic food items in stores; availability of food and drink categories in restaurants		Survey instruments (one for store, one for restaurant)	9 categories of basic food items (stores), 9 categories of foods (restaurants), 10 categories of drinks (restaurants)	Conceptualization of survey instruments was informed by a previous instrument, was done in collaboration with local collaborators. Instruments were pretested for wording and content and then finalized for pilot testing in socioeconomically diverse neighborhoods, score = 3

### Characteristics of measures/instruments assessing availability and accessibility

Table 1 (columns 4–6) shows the characteristics of measures/instruments included. Different types of instruments were used. Over half of the studies included self-report questionnaires; other types of instruments included were: observational tools [36, 38, 40, 43], checklists [35, 42], inventories [30, 37] and a telephone interview [39]. The number of items included in the final instruments varied significantly between studies. Some studies used broader categories of foods and/or drinks with subcategories although reliability and validity were in some cases reported at the food category level only. For example, availability of different types of fruits was assessed by Hearst et al.; reliability was however reported at the category level (i.e. fruit) [35]. In many of the included studies, included instruments also measured other correlates of dietary behaviors; some also included measurements of the physical activity environment.

### Conceptualization of availability and accessibility

With regards to availability, measured in 18 studies, participants were either asked to report whether a certain type of food/drink was present at the time of data collection by selecting options such as yes or no, or by reporting the usual presence of foods. In some studies, the participants were asked whether the food item of interest was served/offered or sold in specified places, e.g. canteens, vending machines etc. In three studies, operational definitions of availability were provided. In the study by Izumi et al., fruits and vegetables were considered to be available if a single portion of the item was present in the store in a ready-to-eat form; for some items, if at least one variety met the criteria, the item was considered available [42]. Nepper et al. defined availability as the existence of food in different locations regardless of whether it is readily visible or accessible to the child [37]. Boles et al. defined availability as whether food is physically located within the home [38]. In the study by Petty et al., the scale assessing availability of fruits and vegetables at home using parental report included items regarding parental consumption [34].

Accessibility was also defined in different manners in the four studies with measures of accessibility. Hearst et al. measured accessibility by exploring whether the food items were present on the kitchen counter or visible when opening the refrigerator door [35]. Nepper et al. used the following definition for accessibility: “a food that is retrievable, ready to eat, or in a location where it is easy for a child to reach it” [37]. In the study by Boles et al. accessibility was defined as whether the child could reach the food [38]. Accessibility was measured in terms of beliefs about family’s affordance of food items; ability to eat everything in reasonable amount and ease of access to a variety of food items in the study by Bennaroch et al. [32].

### Item development and refinement including quality assessment

Table 1 (column 7) provides details of the item development and refinement for each included study. Many of the included studies provided a clear description of how the items were chosen or developed and followed a systematic step in item development and refinement. Twelve of the included studies received the maximum quality score of 4 for item development and refinement [30–33, 36, 38, 39, 41–43, 46, 48], 6 studies received a score of 3 [35, 40, 44, 45, 47, 49] and two studies received a score of 2 [34, 37]. Methods used in the development or refinement of items included using or building on available instruments [30, 34, 36–38, 41, 45, 46, 49], literature review [31, 32, 39, 41, 42, 47], expert opinion [30–33, 36, 38, 39, 46, 48] and use of qualitative methods [31]. Several studies combined two or more of these methods.

### Assessment of reliability and validity

#### Reliability assessment

Table 2 presents the results of reliability analysis in the included studies. Reliability assessment was conducted in 14 studies. Seven studies assessed test-retest reliability [31, 33, 34, 37, 44–46]. The gap between the test and retest was 2 weeks in three studies [31, 33, 34], 1 week in two studies [37, 45] and 2–4 weeks in one study [44]. Test-retest reliability was almost perfect in three studies [31, 33, 34]; it was moderate to substantial for all [44, 45] or most [37] items in the other studies. Test-retest reliability was slight to substantial in the study by Ward et al. [46], with most items having intra-class correlation (ICC) < 0.40 for 1-day ICC; 4-day ICC showed moderate to almost perfect reliability except for one item.

**Table 2:** Psychometric properties of measures included in the review

Author, year, country	Construct, i.e. availability or accessibility	Internal consistency reliability	Inter-rater reliability	Test-retest reliability including timing between measurements	Construct validity	Criterion validity including comparison instrument/method
Boles et al., 2014, US [30]	Availability		Kappa 0.74-0.84 between independent raters			Parental reports compared to direct observation by trained independent raters - lower value for kappa range varied between 0.029 and 0.336 and upper value was 1.00, 62/131 items found to have kappa less than 0.61 and were removed % agreement varied between 92% and 100% for food categories for which it was computed (*n* = 4)
Dewar et al., 2012, US [31]	Availability	Chronbach’s alpha: 0.79, factor loadings (0.44–0.86)		ICC (measurement 2 weeks apart): 0.81 (0.75–0.86)	Confirmatory factor analysis performed with fit indices that were a good or exact fit of the hypothesized model - RMSEA-0.00, CFI-1.00, GFI-1.00, AGFI-0.99	
Benarroch et al., 2011, Spain [32]	Accessibility	Factor loadings of 0.40–0.68			Correlations with dietary behaviors: 0.085 (cheese)–0.248 (vegetables)	
Vyduna et al., 2016, US [33]	Availability	Chronbach’s alpha 0.71		Pearson r (2 weeks apart): 0.80		
Petty et al., 2013, Brazil [34]	Availability	Chronbach’s alpha:0.69 Factor loading 0.70–0.78		Pearson r (2 weeks apart): 0.80	Convergent validity using pearson r: 0.60 (other parent living with the family was asked to answer questions according to how they believed their partner answered the questionnaire previously) significant associations between availability measure and the consumption of fruits, vegetables, soft drinks and sweets (B = -0.92-6.23 for association with weekly frequency of consumption of foods)	
Hearst et al., 2012, US [35]	Availability and accessibility					Criterion validity assessed comparing family reports with those of staff scores kappa for availability: 0.16 to 0.85 (2 items with kappa <0.3); spearman r: 0.20–0.88 (3 items with r <0.3) kappa for accessibility ranged from 0.26 to 0.51 (2 items with kappa <0.3); spearman r ranged from 0.25 to 0.52 (3 items with kappa <0.3), Kappa for obesogenic food availability score was 0.57, spearman r was 0.78
Rimkus et al., 2013, US [36]	Availability		Average kappa was 0.84, proportion of overall agreement was 0.95, ICC was 0.82 for product availability, 52 of the measures (93%) had a kappa ≥0.61, 2 measures had kappa <0.4			
Nepper et al., 2014, US [37]	Availability and accessibility	HFA instrument: for healthy and unhealthy foods and beverages: chronbach’s alpha 0.94 for healthy, 0.91 for unhealthy and 0.90 for total items; for fruit and vegetable items alpha was 0.82 and 0.80. HFES instrument: chronbach’s alpha for healthy and unhealthy food items: 0.83		HFES only (availability), 1 week apart: ICC for unhealthy foods and beverages: 0.79–0.96, ICC for healthy foods and beverages: 0.07–0.93 (all except one item had ICC >0.4)		HFA instrument only: assessed comparing parental report to those of research staff. healthy and unhealthy foods and beverages: for availability, kappa 0.08–1.00, 2 items had kappa <0.3; for accessibility, kappa -0.02 to 1.00, 3 items had kapp <0.3; for fresh fruits, kappa ranged from 0.41–1.00 for availability and 0.38–1.00 for accessibility; for fresh vegetables, kappa ranged between 0.42 and 1.00 for availability and between 0.24 and 1.00 for accessibility, 2 items had kappa <0.3
Boles et al., 2013, US [38]	Availability and accessibility		For healthy and unhealthy foods and beverages: kappa ranged between -0.07 and 1.00, 4 items had kappa values less than 0.60 for availability; kappa ranged between -0.07 and 1 for accessibility, with 1 item with kappa less than 0.60; items with inadequate kappa were subsequently removed; fresh fruits and fresh vegetables: all items had kappa values >0.60		HHE instruments were examined between obese and non-obese children: healthy and unhealthy food items: there was no difference between groups; fruits and vegetables: families of obese preschoolers were significantly less likely to have fresh vegetables accessible in the home compared with healthy weight families; families of obese preschoolers were significantly less likely to have fresh vegetables available compared with healthy weight families;	
Nathan et al., 2013, Australia [39]	Availability					Results compared to observations made by pre-service teachers Percent agreement ranged from 52 to 95 (3 items with % agreement <60), kappa ranged from 0.02 to 0.9, with 13/26 food items having kappa values >0.6, 6 items had kappa <0.3; food sold through fundraisers had higher kappa values than food sold in canteens; no vending machines were reported in the schools, so percentage agreement and kappa scores were 100% for these items
Lee et al., 2014, US [40]	Availability					Compared to direct observation by trained observers (based on 175 meals served, snack consumption of 528 children), for weekly OSNAP-OPAT, r = 0.43 (whole grains) - 0.84 (fruit and vegetable), for daily OSNAP-OPAT, r = 0.32–0.66
Dodds et al., 2014, Australia [41]	Availability					Compared to direct observation by trained research assistants; kappa 0.45 to 1.00; percentage agreement 73% to 100%
Izumi et al, 2014, US [42]	Availability		Kappa values ranged from 0.48 to 1.00, two items had kappa of < 0.60			
Krukowski et al., 2011, US [43]	Availability		% agreement values ranged from 0.38 to 0.99 for school lunch menu assessment with 1 value <0.60, and from 0.41 to 0.95 for observation of foods offered in cafeteria during lunch, with 1 value <0.60			
Ding Ding et al., 2012, US [44]	Availability	Chronbach’s alpha ranged from 0.60 to 0.75 for adolescent report, 0.65 to 0.73 for parent reports for adolescents and from 0.40 to 0.67 for parent report for children (1 item had alpha less than 0.6)		ICC (measurement 2–4 weeks apart) ranged from 0.47 to 0.58 for adolescent report, 0.72 to 0.78 for parent reports for adolescents and from 0.70 to 0.88 for parent report for children	Association with fruit and vegetable intake using partial correlations: -0.18–0.31 for adolescents, -0.17 to 0.27 for parent report for adolescents and 0.15 to 0.34 for parent report for children	
Singh et al., 2011, six European countries (Belgium, Greece, Hungary, the Netherlands, Norway, Spain) [45]	Availability			1 week apart, for availability of fizzy drinks or fruit squash ICC = 0.74, fruit juice ICC = 0.67, and breakfast products at home ICC = 0.42	Face-to-face interviews used: for availability of fizzy drinks or fruit squash ICC = 0.52, fruit juice ICC = 0.57 and breakfast products at home ICC = 0.25, % agreement 80	
Ward et al., 2015, US [46]	Availability			1 day ICC varied between 0.06–0.55, with all values for beverages <0.40 and 1 value for beverages being <0.40. 4 day ICC varied between 0.20 to 0.83, with only 1 value being lower than 0.4		Criterion validity with comparison using obervation by trained data collectors over 4 days, r: 0.25–0.85, with 1 value being less than 0.3
Anzman-Frasca et al., 2015, US [47]	Availability					Criterion validity assessed comparing the program leaders’ responses to observations (2 visits) by trained research assistants and trainers in the first sample and by using digital photography in the second sample. % agreement ranged from 61.5 (water) to 93.9 (sweetened beverages), kappa ranged from 0.08 (sweet snacks) to 0.62 (milk), with 4 items having kappa values less than 0.3. Spearman r = 0.13 (water)-0.56 (salty snacks), only 1 item with r < 0.30
Fulkerson et al., 2012, US [48]	Availability					Criterion validity assessed by comparing results to those of research staff trained to use the screener, kappa ranged between 0.52 and 1 for major food categories served/not served (1 value less than 0.6), kappa ranged between 0.74 and 0.87 for food subcategories served/not served (averaged across foods within same subcategory)
Hua et al., 2014, China [49]	Availability		% agreement between 67.8 and 99.3 for foods in restaurants; between 80.4 and 93.7 for drinks in restaurants; between 86.5 and 98.9 for foods in stores			

Six studies assessed internal consistency reliability [31–34, 37, 44]; an adequate internal consistency was reported in all studies where it was assessed except for 1 item in the study by Ding Ding et al. [44]. Six studies assessed inter-rater reliability [30, 36, 38, 42, 43, 49]. Inter-rater reliability as measured by kappa revealed substantial agreement for all [30] or most [36, 42] of the items included. Percentage agreement was moderate to good-to-excellent for most items in the studies where it was computed [43, 49]. Items with inadequate kappa values (< 0.60) were removed in the study by Boles et al. [38].

#### Validity assessment

All of the studies either provided a clear description of the sample and provided a clear conceptualization of the measure, or provided a clear description of the item development and/or refinement, or used a previously validated measure. Therefore all of the measures were considered as addressing face validity. In addition, nine studies used experts in the development and/or refinement of survey items [30–33, 36, 38, 39, 46, 48]; the measures were therefore considered to have content validity.

Construct validity was assessed in six studies (Table 2). Correlations of the measures with dietary behaviors were assessed in two studies [32, 44] and associations were mostly weak (*r* <0.30). Petty et al. reported significant associations between the availability measure and dietary behaviors (reported as regression coefficients); convergent validity (comparing response with that of the other parent) was acceptable [34]. Acceptable construct validity was reported by Singh et al. (based on ICC and percentage agreement values) [45]. Dewar et al. explored factorial validity using confirmatory factor analysis and found fit indices that were a good or exact fit of the hypothesized model [31]. Boles et al. assessed the discriminant validity of their measure and found differences between obese and non-obese children for the availability and accessibility of vegetables but not for other included foods [38].

Criterion validity was assessed in ten studies (Table 2). The criterion measure was either direct observation by trained research staff [30, 39–41, 46] or comparison with responses reported by research staff trained to use the instrument [35, 37, 48]. In one study, digital photography was used in one sample and direct observation in the second sample [47]. Acceptable validity was achieved for all final items in some studies [30, 40, 41, 48]. In other studies most items achieved adequate criterion validity [35, 37, 39, 46, 47].

#### Responsiveness

None of the included studies included an assessment of the responsiveness of the instrument.

## Discussion

The review aimed to summarize the results of methodological studies exploring the psychometric properties of measures of the availability and/or accessibility of food among youth, and to describe differences in the conceptualization of these correlates across studies. A total of 20 studies were identified and only four assessed accessibility. There were differences in the conceptualization of the correlates between studies, in particular accessibility of food. Different assessments of reliability and accessibility were conducted in the included studies, and most measures were found to have adequate evidence of reliability and/or validity. None of the studies assessed responsiveness.

### Samples included

Determination of sample size for studies of validity and reliability assessment has long been a matter of discussion and no strong consensus exists [50, 51]. However, the sample size of some of the included studies [35, 37] appears small even by the most liberal estimates [22]. Testing these instruments among a larger sample would therefore appear important. It is also important to report information about missing data and participation rate, which was not always the case in the included studies, as these might affect the interpretation of results. Many of the studies included participants or settings with different socioeconomic backgrounds although several studies had fairly homogeneous samples. Two studies focused on low income participants [35, 40], another included participants with a predominantly low SEP [30]. Availability and accessibility of food are known to differ by socioeconomic position [52] and groups with low socioeconomic position often bear a disproportionate burden of overweight and obesity. More testing of instruments in specific socioeconomic subgroups or settings, and in particular in low socioeconomic groups and ethnic minorities, is thus needed. Studies, if powered to do so, could also look at similarities or differences between different socioeconomic subgroups within the same study.

### Conceptualization of availability and accessibility

There were some variations in how availability was conceptualized in different studies, partly dependent on the setting explored. Availability and accessibility of food can be assessed in all the different environments that youth get exposed to (i.e. home, school or early care settings, stores or restaurants). Availability, which refers to the physical presence of food, should be overall conceptualized in the same manner across these settings. For the home environment, this implies that the food is present somewhere in the home; for schools, stores or restaurants, a food is available if sold or offered in these settings. However, further refinements should be made as appropriate to increase the feasibility of use of the measurement instrument as the type and volume of foods in these different settings vary significantly. In this regard, one of the issues that might arise with the assessment of availability is whether one should count all possible varieties of certain foods or whether there is some acceptable minimum number of varieties [53]. This is particularly relevant when environments such as food stores are assessed and when food types with a large number of categories are assessed. A clear definition of availability should be provided in such cases. There were also variations in the conceptualization of accessibility across studies. Accessibility can cover different aspects [15] including location (e.g. fruits and vegetables put on the table vs. the refrigerator), form (e.g. whole vs. sliced fruits) and cost. There is a need to uniformly define what is meant by accessibility of food in order to compare studies and make conclusions about the most important aspects of accessibility that influence dietary behaviors. For example, visibility has been used as a dimension of accessibility [35]; it has also been defined as a construct by itself, separate from accessibility [37]. Food could indeed be visible but not reachable. Even though visibility can increase a child’s attention to food, its effect might be different to accessibility which included retrievable and reachable food [37] so this separation of constructs appears justified. Applying a theory or framework to identify relevant items to be included in the measurement instruments and conducting thorough analyses (e.g. factor analyses) to refine items when multiple items are used appears important. Another way to develop a thorough conceptualization of these concepts is the use of expert opinion, which was used in several studies, as well as the use of qualitative methods, which can also help generate theory. Due to the few studies that assessed accessibility of food, this review cannot provide further clarity to the conceptualization of accessibility.

### Reliability and validity

In most of the included studies, there was a satisfactory evidence of internal consistency reliability which could among other things reflect the thorough process of development of items via the use of literature, expert opinion, theory and previously validated instruments. Evidence of test-retest reliability was also largely satisfactory although some variation between items was observed. Although there are specific cut points for the identification of reliability values that are acceptable, the item and the type of data collected should also be taken into consideration. Parameters of reliability such as the ICC are affected by inter-individual variation, being low in homogeneous samples [22]. This reflects the importance of assessing the ICC of an instrument in the same or comparable population to the one where it will be used [22]. The low reliability values for the measures of the availability of some foods might also indicate factors such as the day-to-day variability in the availability of those foods and not necessarily errors in reporting from the side of participants [46]. In the present review, the time gap between the two administrations for establishing test-retest reliability varied between studies, with a time gap of up to 4 weeks used. Although there is no single recommended time gap for the administration of instruments while assessing test-retest reliability, this time gap can have an effect on reliability estimates.

Previous reviews of measurement instruments of correlates of energy balance-related behaviors among youth have concluded that there was a limited assessment of criterion validity of measures, partly due to a lack of a gold standard [18, 23, 54, 55]. The assessment of reliability and validity is influenced by different factors including the purpose for which the instrument is used. There are two pathways through which the food environment can affect behaviors: through perceptions of the environment or through actual physical characteristics of the environment [20]. The choice of measurement instrument and the type of validity assessed depends on which of these two aspects is explored. Perceptions are commonly measured using self-report instruments (e.g. surveys, interviews) [20]. The objective environment is best assessed via methods such as direct observation or documentation [20]; or via self-report instruments validated using methods such as direct observation (i.e. criterion methods). Several of the studies included in this review which developed measurement instruments of the availability of food assessed criterion validity. The criterion measures were direct observation by trained research staff, instrument filled in by trained staff and digital photography.. This assessment of validity is very valuable as it reflects the ability of these measures to provide a good assessment of the actual presence (i.e. availability) of foods, and measures with such evidence of validity [30, 35, 37, 39–41, 46–48] should be recommended for use in future studies, or for further testing in other samples. However, criterion validity would not be useful when the interest is to explore perceptions. Unlike availability, which reflects the actual presence of foods, accessibility can involve the perception of whether what is available is actually easily accessible. For example, the assessment of the ease with which food can be consumed as well as the cost related to food, which might be components of food accessibility, are prone to differences in perceptions, making it difficult to use any criterion measure. In these cases, other measures of validity such as construct validity are more relevant, in particular when thorough empirical or theoretical information is used to define hypothesized associations. Construct validity was assessed in some studies included in this review with associations that varied from weak to strong. The weak or absent associations might reflect a genuine lack of association of the measures with the chosen outcome, in particular if the hypothesized associations were not based on thorough evidence or theory; they might also reflect lack of validity of the measures, of the hypothesized determinant (availability or accessibility) or of the outcome.

### Responsiveness

Although the present review is limited to methodological studies, none of the included studies measured responsiveness. The lack of assessment of responsiveness is of concern, as evidence about sensitivity to change of such instruments is important, in particular for intervention studies. However, assessment of responsiveness requires time and resources as follow-up of participants is required.

Direct comparison of the psychometric properties of measures of food availability and accessibility within and across reviews is complicated due to the differences such as the type of measures, number of items, food types included and the age group targeted. Previous reviews have documented the presence of measures of food availability and accessibility among youth with one or more satisfactory psychometric properties tested in specific samples [18–20]. The present review similarly identified several systematically developed measures with adequate psychometric properties including several measures with criterion validity. Some of the survey instruments/measures were culturally adapted and new for the specific context they were used in. These include the instrument by Hua et al. used to assess the Chinese food environment [49] and the instrument by Petty et al. which represented a translation of the parental mealtime action scale into Portuguese and used in a Brazilian sample [34]. Hearst et al. similarly refined and validated an existing home food inventory for use in specific cultural groups (Spanish- and Somali- speaking low income families) [35].

### Other considerations

Fluctuations in availability that might occur because of factors such as seasonal variations and shopping routines when actual availability is assessed (e.g. inventories) are important to consider. Surveys conducted directly after food purchase will give estimates that are different from surveys conducted at a later time point. This might be more problematic in low income settings where availability might be even more variable [53]. Different methods can be used to address this problem. One is to measure usual availability or availability over a given period. This was done by Nepper et al. who developed both a one-time assessment tool and a 30-day home environmental survey instrument [37]. The second option is to conduct inventories immediately after shopping episodes. Recording items on repeated occasions and adjusting data for the number of days since shopping was last done have also been suggested [19].

Some of the measures included, as evidence by the number of items included (Table 1), are lengthy and might require considerable time for participants to complete; their feasibility might thus be limited for some studies.

### Strengths and limitations of review

This review was limited to methodological studies, as the main aim was to include instruments where the process of development as well as validity and reliability evidence were described in detail, and to focus mainly on newly developed instruments. It is however possible that there are measures with at least some evidence of test-retest reliability and/or construct validity that were missed by excluding non-methodological studies. This includes studies that might have used the instruments included in this review. The search strategy may not have captured all relevant articles. The strengths include the systematic process used in the identification of studies, the assessment of quality of processes for instrument development and evaluation, as well as the use of two independent researchers for the extraction of data and quality assessment.

Strengths and weaknesses of available instruments and recommendations for further research Several of the instruments in the included studies represented improvements from previous instruments; they were developed to increase feasibility of use and to improve use in different population groups as well as different settings (including diverse school types). While self-report questionnaires were predominant, as was also the case with older instruments assessing availability and accessibility of food among youth [18, 19], some observational tools were also developed and tested in schools, afterschool programs, stores and at home. While observational tools using trained researchers might provide the most objective assessment of the food environment, they might be costly and hence using instruments that can be filled in by parents or available personnel at school or daycare is important [46]. There is a strong focus on the home environment in relation to the dietary behaviors of youth [9]. In line with that, methodological studies including measures of home availability and accessibility of food appeared predominant. As the obesogenic environment is being increasingly recognized as a driver of the obesity epidemic, including among youth, more studies addressing availability and accessibility outside the home are called for, although some good measures do already exist. This is particularly true for the neighborhood food environment such as stores that might be important sources of unhealthy food, in particular among adolescents. Few studies have focused on accessibility therefore more methodological studies looking at measures of food accessibility are needed, with a focus on operationalization of the construct in addition to testing psychometric properties. Studies in countries other than the US and in particular in non-Western settings and studies looking at particular population subgroups are also needed.

## Conclusion

This review identified 20 methodological studies exploring the psychometric properties of measures of availability and/or accessibility of food related to youth. The studies included several food items, and most studies followed systematic steps in the development of the measures. More than half of the measures focused on the home environment, and only four studies included measures of accessibility. The majority of the measures had satisfactory evidence of reliability and/or validity. However, there were variations in the conceptualization of the correlates, in particular accessibility. More studies including measures of accessibility and addressing its conceptualization are needed. More testing of some of these measures in different population groups is also warranted, as is the development of measures of food availability and accessibility in the broader environment such as the neighborhood food environment.

## Abbreviations

ICC: Intra-class correlation
PRISMA: Preferred Reporting Items for Systematic reviews and Meta-Analyses
